# Exploring and enhancing midwives’ professional autonomy: Embarking on a journey of empowerment for midwives globally

**DOI:** 10.18332/ejm/172426

**Published:** 2023-10-24

**Authors:** Joeri Vermeulen, Ronald Buyl, Maaike Fobelets

**Affiliations:** 1 Department of Health Care, Erasmus Brussels University of Applied Sciences and Arts, Brussels, Belgium; 2 Faculty of Medicine and Pharmacy, Department of Public Health, Biostatistics and Medical Informatics Research group, Vrije Universiteit Brussel, Brussels, Belgium; 3 Brussels Institute for Teacher Education, Vrije Universiteit Brussel, Brussels, Belgium

**Keywords:** midwives, autonomy, midwifery, professionalization, professional autonomy, midwifery autonomy

In the ever-evolving landscape of healthcare professions, the importance of professional autonomy has been widely recognized, with positive impacts on job satisfaction, sense of belonging, and subjective well-being^1-3^. While literature is inconclusive about whether midwives themselves want to be more autonomous^4-6^, a WHO report confirmed that midwives worldwide seek more professional autonomy^7^. Here, we delve into the diverse dimensions of Belgian midwives’ professional autonomy. Through this exploration, we illuminate persistent knowledge gaps. As we traverse this domain, we extend an invitation to the global maternity care community, urging reflection on the interplay between autonomy, aspiration, and external influences. This journey is dedicated to advancing empowerment for midwives worldwide.

The phenomenon of midwifery autonomy is complex, intertwined with contextual influences, societal perceptions, policy frameworks, and educational paradigms. A study exploring the state of professionalization of midwifery in Europe recommended more in-depth studies on midwives’ autonomy and maternity care culture in individual countries^8^. The authors chose to explore Belgian midwives’ professional autonomy, as the status of their professional autonomy was undisclosed while insights from stakeholders are lacking. This research could have a positive impact on the professionalization of midwifery, not only in Belgium but also in other countries facing similar challenges.

To gain a comprehensive understanding of Belgian midwives’ professional autonomy across all regions and professional domains, five studies were conducted:

1. Our journey commenced with an analysis of relevant policy and academic texts, underpinned by Greenwood’s sociological criteria for a profession. As such, the state of the professionalization of midwifery in Belgium was described. We identified documented challenges spanning societal, structural, and professional domains. Recommendations to advance the midwifery profession involve increasing public awareness, implementing comprehensive midwifery practices, and enhancing professional autonomy through collaborative endeavors among midwifery organizations, women’s groups, maternity care professionals, and policy-makers^9^;
2. The forging of a consensus definition of ‘midwifery autonomy’, using a modified Delhi study with 27 content experts, was an ambitious stride, fostering a deeper grasp of this concept^10^. We established a communal definition of midwifery autonomy in Belgium, comprising 15 components related to midwives’ work content, professionalism and relationship with others;
3. Through meticulous inquiry, a subsequent descriptive observational study among 312 midwives revealed nuanced perceptions from midwives. Amidst this diverse tapestry of perspectives, notable disparities emerged between hospital-based and primary care midwives, as well as across various regions. Furthermore, the study echoed midwives’ desire for heightened professional autonomy, coupled with a strong wish for societal recognition and respect in the realm of maternity care. Yet, a complex interplay emerged – the intricate interposition of perceived autonomy and latent fears of liability^11^;
4. Additionally, through heterogeneous virtual focus group interviews (n=3), using a qualitative approach, maternity care stakeholders (n=27) views on midwives’ professional autonomy were identified. Stakeholders, including health professionals, policy advisors, hospital managers and service-users emphasized vital prerequisites for professional autonomy, including education, continuous professional development, competence, experience, and collaboration. Furthermore, stakeholders stressed the emergence of an integrated maternity collaborative framework and advocated for stringent regulations to uphold care quality and fortify midwives’ autonomy^12^; and
5. Finally, through homogeneous in-person focus group interviews (n=3), using a qualitative approach, the experiences and understanding of midwifery autonomy among final-year midwifery students was explored. Students emphasized the need for supportive learning environments, collaborative practice and continuous professional development to promote midwifery autonomy. In students’ views, cultivating safe and supportive learning environments, may contribute to midwives’ professional autonomy, ultimately improving maternal and new-born health.

Our research illuminated barriers compromising the autonomy of Belgian midwives, categorized them through the lens of Greenwoods’ professionalism criteria^13^. With these insights in hand, a clear call for action emerged, urging a focus on recognized authority, to prevent midwives from being undervalued, underutilized, and underpaid. Aligning regulation and education with the International Confederation of Midwives’ standards, promotes midwives’ professional autonomy worldwide^14^. Additionally, strengthening professional leadership in midwifery, from local to international levels, is essential. This entails developing leadership within the midwifery profession and forging partnerships across political, societal, and inter-professional domains. Such efforts are key to enhancing midwifery autonomy and elevating its global influence^15^.

Recommendations to enhance midwives’ professional autonomy encompass prioritizing midwife-led continuity of care, fostering interprofessional collaboration, tailoring continuous professional development, increasing public awareness, and advocating for policy changes. The trajectory to empowerment necessitates a metamorphosis, cultivating environments where autonomy flourishes. Our research provided valuable insights in how to promote midwives’ professional autonomy. Nevertheless, it is important to acknowledge certain limitations in this study, including the inherent subjectivity in our interpretations and the potential challenge of generalizing findings beyond Belgium.

We extend an open invitation to engage, initiate dialogue, and foster alliances across borders and maternity care disciplines. As we unite to embrace professional autonomy, recognition, and collaborative excellence, the journey that was embarked upon by Belgian midwives resonates as an embodiment of empowerment, shaping the trajectory of midwifery, not only within Belgium but also on the global stage.
